# Ion-Based Proteome-Integrated Solubility Alteration Assays for Systemwide Profiling of Protein–Molecule Interactions

**DOI:** 10.1021/acs.analchem.2c00391

**Published:** 2022-05-04

**Authors:** Christian M. Beusch, Pierre Sabatier, Roman A. Zubarev

**Affiliations:** † Chemistry I, Department of Medical Biochemistry and Biophysics, Karolinska Institute, Stockholm 17177, Sweden; ‡ Department of Pharmacological & Technological Chemistry, I.M. Sechenov First Moscow State Medical University, Moscow 119146, Russia; § The National Medical Research Centre for Endocrinology, Moscow 115478, Russia

## Abstract

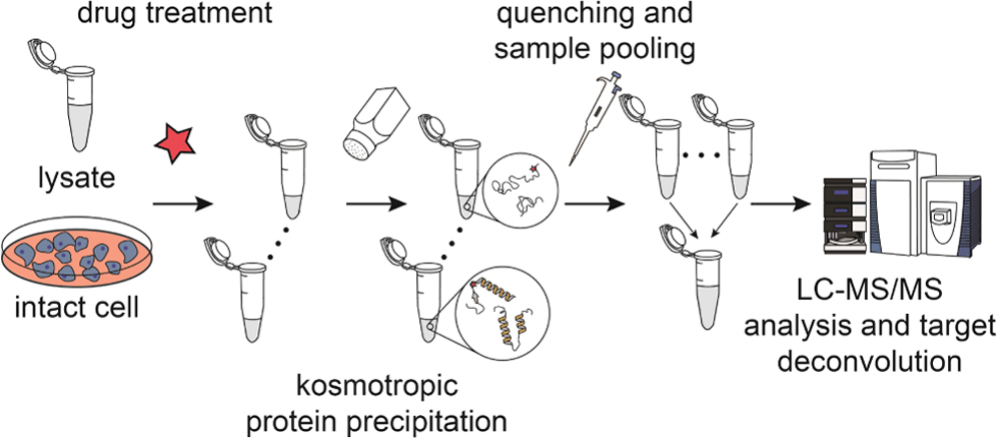

Unbiased drug target engagement deconvolution and mechanism of action elucidation are major challenges in drug development. Modification-free target engagement methods, such as thermal proteome profiling, have gained increasing popularity in the last several years. However, these methods have limitations, and, in any case, new orthogonal approaches are needed. Here, we present a novel isothermal method for comprehensive characterization of protein solubility alterations using the effect on protein solubility of cations and anions in the Hofmeister series. We combine the ion-based protein precipitation approach with Proteome-Integrated Solubility Alteration (PISA) analysis and use this I-PISA assay to delineate the targets of several anticancer drugs both in cell lysates and intact cells. Finally, we demonstrate that I-PISA can detect solubility changes in minute amounts of sample, opening chemical proteomics applications to small and rare biological material.

## Introduction

Besides binding to their target proteins, small-molecule drugs often exhibit off-target effects by interacting with other proteins, which can confound the specific target inhibition or even lead to adverse effects in the clinic.^1−3^ To minimize these issues, it is therefore important to determine the target landscape of every candidate molecule in an early stage of drug development. With that purpose in mind, a series of analytical methods have been developed over the years. Whereas early techniques required *a priori* knowledge of the binding partners or were based on the target enrichment strategies employing tagged drug molecules,^4−8^ more recent approaches do not demand target knowledge or chemical modification of the drug.^9−11^ These unbiased approaches are based on the detection of an alteration in physicochemical properties of the target proteins upon binding a small molecule in a complex matrix containing a multitude of noninteracting proteins. Most prominently, thermal proteome profiling (TPP),^9^ the untargeted and proteome-wide version of the Cellular Thermal Shift Assay (CETSA),^4^ relies on the concept that a small molecule binding to a protein alters the thermal stability of the latter. To assess protein thermal stability, samples are treated at different temperature points, at which conditions partial protein denaturation is induced. After isolating and analyzing the soluble proteome part, sigmoidal “melting curves” are constructed, therefrom the melting temperature (*T*_m_) of each protein is calculated. A melting temperature shift (Δ*T*_m_) in the presence of the drug indicates drug binding to the protein when TPP or CETSA is performed in cell lysates. In the assays made in intact cells, Δ*T*_m_ can also reveal proteins downstream of the target.

TPP has shown great value not only in drug target deconvolution but also in fundamental molecular biology. This is due to the fact that most alterations in protein primary, secondary, and higher-order structures, whatever their causes, shift the protein melting curves and Δ*T*_m_. Therefore, TPP is now widely used in diverse applications, such as the study of protein–protein interactions,^12^ metabolite–protein interactions,^13,14^ determination of the enzyme substrates,^15^ and studying cellular mechanisms.^16−20^

Despite the versatility of temperature-based protein precipitation methods, they have several limitations. One of them is that not all proteins lose their solubility with increasing temperature. Another limitation is that the melting curves are not always of the presumed sigmoidal shape, which complicates *T*_m_ determination.^21^ Additionally, thermal-based protein precipitation methods cannot be applied when thermally labile systems are under study.^22,23^ Thus, alternative, isothermal methods of protein precipitations are highly desired. Consequently, several groups developed approaches probing the protein solubility by other means, such as Solvent-Induced protein Precipitation (SIP)^24,25^ and Mechanical Stress Induced Protein Precipitation (MSIPP).^26^ However, these methods seem to be solely applicable to lysate experiments and unlike TPP cannot be used in intact cells. Additionally, inducing protein precipitation in SIP by adding organic solvent at different concentrations precludes sample pooling by simple combining. The samples treated at different conditions need in these approaches to be individually centrifuged and processed for collection of the supernatant, which limits the throughput and sensitivity of the analysis.

In this work, we improve the above deficiencies by introducing a fast isothermic approach that explores ion-induced precipitation. It is well known that some ions have the ability to change protein solubility, and thus, proteins can be “salted out” by such kosmotropic agents as well as they can be “salted in” by chaotropic ions.^27^ These cations and anions can be ordered in the Hofmeister series based on their effect on protein solubility.^28^ Whereas protein thermal unfolding is assumed to be mainly caused by disruption of hydrogen bonds and nonpolar hydrophobic interactions,^29,30^ the mechanism behind the Hofmeister series is still debated but seems to result from changes in specific electrostatic interactions between solvated ions and proteins.^27,28,31^ By combining ion-based protein precipitation with our previously introduced Proteome-Integrated Solubility Alteration (PISA) assay,^32^ we created a novel Ion-based Proteome-Integrated Solubility Alteration (I-PISA) method. Here, we demonstrate that I-PISA is able to identify known targets of various drugs not only in lysates but also in intact cells. Drug molecules are actively imported to and exported from the cells as well as get metabolized inside the cell, with the resulting metabolites often binding target proteins with greater affinity than the original drug. Therefore, the ability to probe target engagement in intact cells is irreplaceable. Furthermore, a direct comparison of the I-PISA assay with the conventional T-PISA assay showed high complementary of the two precipitation approaches, with examples found when the solubility changes have opposite directions. Additionally, we were able to apply I-PISA to samples with lower protein amount than we could do with T-PISA, likely due to the difference in seeding properties of the precipitating agents. These features of I-PISA can open novel opportunities in both drug discovery research and addressing fundamental molecular biology questions.

## Materials and Methods

Detailed experimental information including cell culture, cell lysate preparation, proof-of-principle samples, proteomic sample preparation, offline high pH fractionation, liquid chromatography–mass spectrometry (LC–MS/MS) methods, and data analysis, including curve fitting for TPP and IPP data, are provided in the Supporting Information PDF file.

### I-PISA with Dilution of Kosmotropic Ions

The A549 lysate was treated with 10 μM methotrexate (MTX) or an equal amount of dimethyl sulfoxide (DMSO) for 15 min at room temperature (RT). Samples were aliquoted into polymerase chain reaction (PCR) tubes and incubated with the corresponding concentration of CuCl_2_ (190–370 μM, step of 20 μM). After 10 min, kosmotropic ion concentration was equilibrated to the lowest one by 50 mM 4-(2-hydroxyethyl)-1-piperazineethanesulfonic acid (HEPES) buffer containing 150 mM NaCl at pH 7.4 supplemented with a protease inhibitor. Samples were pooled and aggregated proteins were removed by ultracentrifugation with 100,000*g* for 20 min at 4 °C. The supernatant was collected, the protein concentration was determined by bicinchoninic acid (BCA) (Thermo Fisher Scientific), and samples were stored at −80 °C until prepared for LC–MS/MS analysis.

### I-PISA Experiments in Lysates

Protein lysates were treated with the corresponding drugs at 10 μM final concentration or an equal amount of DMSO for 15 min at room temperature. Samples were aliquoted into PCR strips that were treated with an increasing concentration of ZnCl_2_ ranging from 150 to 600 μM (steps of 50 μM). Samples were vortexed and incubated for 10 min at room temperature (RT). Thereafter, the precipitation reaction was quenched with a 2× molar excess of Na_2_HPO_4_. After vortexing, samples were pooled and aggregated proteins were removed by ultracentrifugation at 100,000*g* for 20 min at 4 °C. The supernatant was collected, the protein concentration was determined by the BCA assay (Thermo Fisher Scientific), and samples were stored at −80 °C until prepared for LC–MS/MS analysis.

### T-PISA Experiments in Lysates with Dasatanib

The same treated lysates as for the corresponding I-PISA sample were used. After samples were aliquoted into PCR tubes, they were heated in a Mastercycler X50s (Eppendorf) for 3 min in a temperature range between 48 and 59 °C; thereafter, they were incubated for 4 min at RT before being placed on dry ice. Samples were pooled and aggregated proteins were removed by ultracentrifugation, in the same centrifugation cycle as the corresponding I-PISA samples.

### I-PISA Experiments in Intact Cells

K562 cells were grown to exponential growth and aliquoted at a concentration of 1.5 × 10^6^ cells/mL. Cells were treated with the corresponding drugs at 2 μM final concentration or an equal amount of DMSO and incubated for 2 h in a cell incubator. Cells were collected by centrifuging for 3 min at 320*g* and washed twice with 50 mM HEPES buffer containing 150 mM NaCl at pH 7.4 before being resuspended in the same buffer but containing Halt protease inhibitor (Thermo Fisher Scientific). Cells were then aliquoted into PCR strips and treated with an increasing concentration of ZnCl_2_ ranging from 150 to 600 μM (steps of 50 μM). After mixing, samples were lysed in the corresponding ZnCl_2_ concentration by three freeze–thaw cycles in liquid nitrogen. Kosmotropic ions were quenched by a two times molar excess of Na_2_HPO_4_. After 10 min, samples were pooled and aggregated proteins were removed by ultracentrifugation at 100,000*g* for 20 min at 4 °C. The supernatant was collected, the protein concentration was determined by the BCA assay (Thermo Fisher Scientific), and samples were stored at −80 °C until prepared for LC–MS/MS analysis.

### Proteomic Profiling by TPP and IPP

For proteomic analysis, the cell lysate was aliquoted in PCR strips in two replicates for treatment at eight temperature or concentration points. For TPP, samples were heated for 3 min in a Mastercycler X50s at the following temperature points: 37, 42, 46, 50, 54, 58, 62, and 67 °C; thereafter, they were incubated for 4 min at RT before being put on dry ice. IPP samples were treated with ZnCl_2_ (individual concentration points were: 0, 150, 250, 350, 450, 550, 650, and 800 μM) for 10 min at RT. Individual samples were centrifuged at 100,000*g* for 20 min at 4 °C. Soluble fractions were transferred to new tubes, and the protein concentration at the lowest temperature (TPP) or ion concentration (IPP) was measured. The same volume from each sample was then used for proteomics sample preparation.

### I-PISA with 1 μg of Starting Material

Microscope cover slides (Chance Propper) were washed once with methanol, followed by two washes with Milli-Q water. To reduce protein binding, the cover slides were incubated for 30 min in the fridge with 0.2 μg/μL bovine serum albumin (BSA) (Thermo Fisher Scientific). Excess BSA was removed by three washes with 50 mM HEPES buffer containing 150 mM NaCl at pH 7.4. The K562 lysate was treated with 10 μM MTX or an equal amount of DMSO for 15 min at RT. A sample containing 1 μg of protein was divided into five samples of 200 ng each, which were pipetted onto the cover slides. ZnCl_2_ was added (starting at 100 μM, steps of 100 μM), and samples were carefully pipetted for mixing. After 10 min, kosmotropic ions were quenched by adding a two times molar excess of Na_2_HPO_4_. After 10 min, samples were pooled into low bind tubes (Eppendorf), which were coated with BSA the same way as the cover slides. Samples were centrifuged for 20 min at 20,000*g* at 4 °C to remove aggregated proteins. Samples were reduced with 5 mM dithiothreitol (DTT) for 1 h at RT, followed by alkylation with 15 mM IAA for another hour in the dark. Then, 20 ng of trypsin (Promega) was added, and samples were incubated overnight at RT. Peptide mixtures were acidified with TFA to pH < 3 and cleaned by StageTips. Samples were dried in a SpeedVac (Genevac) and stored at −80 °C.

### Mass Spectrometry

The samples were resuspended in 2% acetonitrile (ACN) and 0.1% formic acid (FA) (buffer A) and injected into an UltiMate 3000 UPLC autosampler or EASY-LC (Thermo Scientific Scientific). The peptides were loaded on a trap column (Acclaim PepMap 100 C18, 100 μm × 2 cm) and separated on a 50 cm long C18 Easy spray column (Thermo Scientific Scientific). All of the chromatographic gradients and the MS settings can be found in the Supporting Information.

### Data Processing and Statistical Analyses

All raw files acquired by data-dependent acquisition were searched on MaxQuant version (2.0.1.0) using the Andromeda search engine.^33^ For the TMTpro-labeled samples, a custom-modified version of MaxQuant was used, recognizing TMTpro as an isobaric label. For peptides search, acetylation of the N-terminal and oxidation of methionine were selected as variable modifications, whereas carbamidomethylation of the cysteine was selected as a fixed modification. Trypsin with up to two missed cleavages was set as protease, and the spectrum was searched against the SwissProt homo sapiens database (20,382 entries). The FDR was set to 0.01 for both peptide and protein identification. For all other parameters, default settings were used. For the raw files acquired using data-independent acquisition, Spectronaut 15.1 (Rubin) was used for identification and quantification of proteins, with the same SwissProt homo sapiens database, and with all other settings left at default.

### Data Availability

The mass spectrometry proteomics data files are deposited to ProteomeXchange Consortium (http://proteomecentral.proteomexchange.org) via the PRIDE partner repository with data identifiers PXD030695 and PXD033081.

## Results and Discussion

### Establishing Ion-Based Protein Precipitation Approach

**Figure 1:**
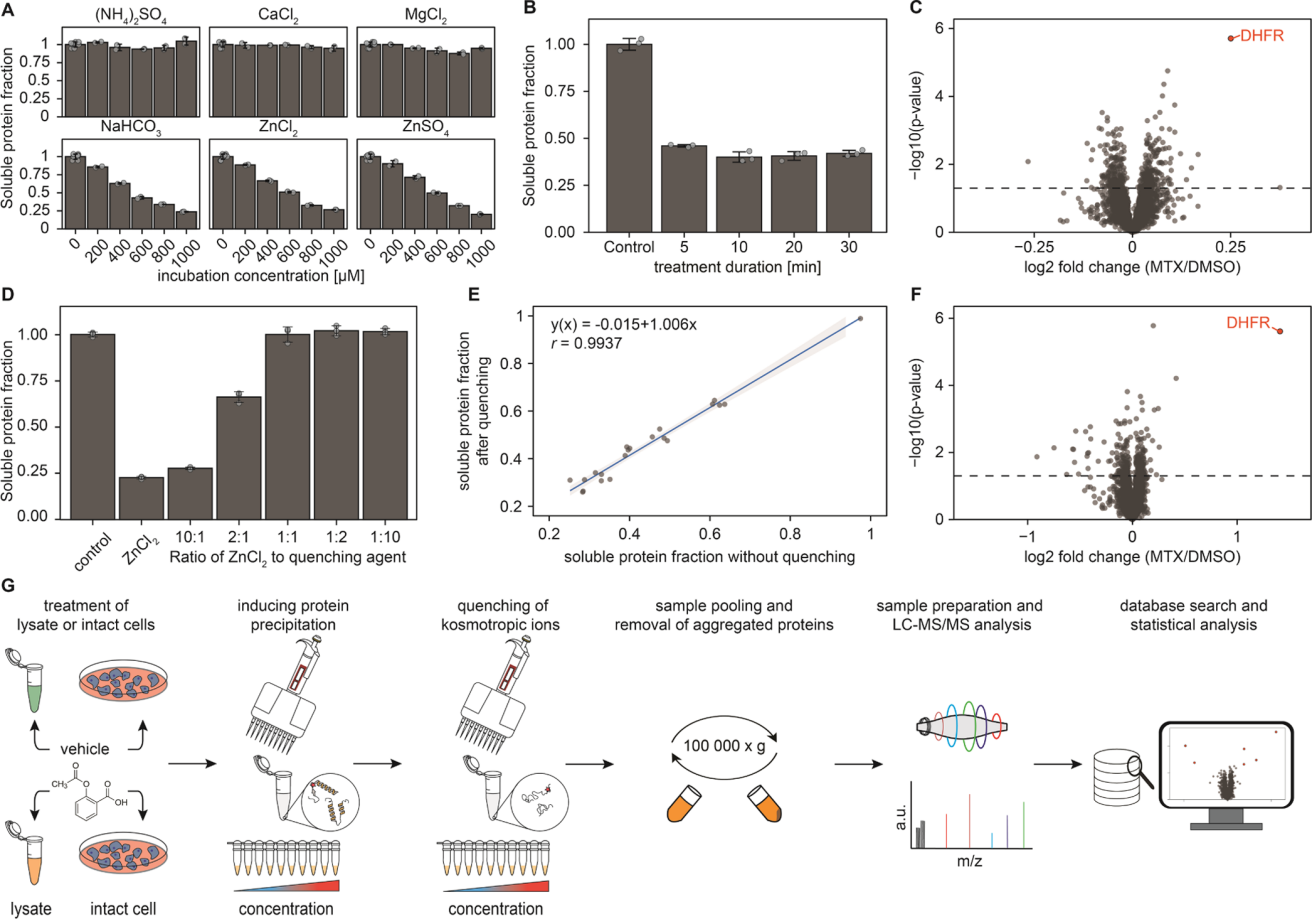
Establishing an ion-based protein precipitation approach. (A) Ions from the kosmotropic range of the Hoffmeister series can precipitate proteins in a concentration-dependent manner out of the complex cell lysate. (B) Protein precipitation by ZnCl_2_ is time-independent after 10 min. (C) Kosmotropic effect of ions can be successfully applied to identify dihydrofolate reductase (DHFR) as a direct target of MTX in lysates among 4119 proteins. (D) Addition to the cell lysate of the already quenched 1 mM ZnCl_2_ salt does not induce protein precipitation when the concentration of the quenching agent is equal to or exceeds that of ZnCl_2_. (E) Quenching of kosmotropic ions by Na_2_HPO_4_ does not induce resolubilization of denatured proteins. (F) Volcano plot of protein solubility alteration in the lysate treated with MTX using quenching before sample pooling, identifying DHFR as the sole directed target (4817 proteins quantified). (G) Schematic diagram of the workflow for the optimized I-PISA experiment with quenching of kosmotropic ions prior to sample pooling.

As most known Hofmeister series are obtained using either individual proteins or simple protein mixtures, in analyzing cell lysates, we could not blindly rely on published tables and started out by testing experimentally lysate-precipitation properties of six salts: (NH_4_)_2_SO_4_, CaCl_2_, MgCl_2_, NaHCO_3_, ZnCl_2_, and ZnSO_4_. Treating the cell lysate by stepwise increasing the concentration to up to 1 mM of these salts followed by centrifugation and measuring protein concentration in the supernatant revealed the concentration-dependent protein precipitation for three out of the six salts (Figure 1A).

Not surprisingly, these three salts contain either anions (HCO_3_^–^) or cations (Zn^2+^) from the kosmotropic part of the Hofmeister series.^28^ Next, we aimed to determine whether protein precipitation due to the kosmotropic ionic effect is time-dependent. For that, we incubated the protein lysate in ZnCl_2_ at 600 μM for 5, 10, 20, and 30 min. While there was a significant change in the protein concentration of the supernatant observed between 5 and 10 min of incubation, there was no significant difference between 10 min and later time points. Thus, we concluded that 10 min of incubation was the optimal duration (Figure 1B). Similar results were obtained when incubating the protein lysate with CuCl_2_, as another example of a salt producing kosmotropic ion from the Hoffmeister series (Supporting Information Figure S1a). From these initial experiments, we chose an incubation of proteins for 10 min as the optimal precipitation conditions.

Based on these initial experiments, we aimed to determine the potential of I-PISA for measuring target engagement; to this end, the A549 lysate was treated for 15 min with 10 μM methotrexate (MTX) known for its prominent target dihydrofolate reductase (DHFR).^34^ After protein solubility was modulated by CuCl_2_, the concentration of kosmotropic ions in each individual sample was equalized by adding corresponding buffer volumes. Next, the individual samples were pooled, as common in a PISA-style experiment, and aggregated proteins were removed by ultracentrifugation. The remaining steps of sample preparation were executed as in a conventional PISA assay, which included collection of the supernatants, protein digestion, TMT labeling, multiplexing, fractionation with reversed-phase high-performance liquid chromatography (HPLC) and analysis by LC–MS/MS.^32^ Statistical analysis of protein abundances with and without the drug present revealed DHFR to be the protein with most significantly altered (increased) solubility, confirming the hypothesis that the ion-based precipitation method can indeed be used to detect protein–small-molecule interactions (Figure 1C).

### Optimization of the I-PISA Assay

While in our initial I-PISA experiment, we used CuCl_2_ to precipitate proteins, eventually, we decided in favor of using ZnCl_2_ for further studies. The main reason for that decision was that protein precipitation with copper happened too abruptly upon reaching a certain critical concentration, which gave less precise data than the smoother precipitation curves provided by zinc salt (Figure S1B). While a reduction in the concentration range of CuCl_2_ would have resulted in a smother protein precipitation curve, a narrower concentration range is more prone to experimental errors.

In a PISA-style experiment where individual concentration points are combined for analysis, straightforward pooling of samples with different salt concentrations will lead to equilibration of the latter, which will cause additional precipitation of proteins from the samples with below-average salt concentrations. One way around this problem is to dilute all samples to the same (lowest) salt concentration and then pool them. Even though this method worked and produced useful results (Figure 1C), we found it unsatisfactory in general due to the large sample volume that it yields. The low protein concentration resulting in the pooled sample negatively affects the protein precipitation rate, necessitating long centrifugation times, while a large volume requires protein preconcentration before proceeding to digestion. Instead of sample dilution before pooling, we found a way to irreversibly “quench” the protein precipitating property of the metal cations by adding to the samples a solution of ions forming an insoluble salt with the kosmotropic ions. After testing different compounds, we identified Na_2_POH_4_ as the optimal quenching agent as it does not precipitate proteins even at high concentrations (Figure S1C) and effectively stops the zinc-induced protein precipitation before I-PISA samples are pooled together (Figure S1D,E). As demonstrated in Figure 1D, addition to the cell lysate of the already quenched 1 mM ZnCl_2_ salt did not lead to protein precipitation if the quenching agent concentration was equal to or exceeded that of ZnCl_2_. Also, addition of Na_2_POH_4_ at a 1:1 ratio with ZnCl_2_ did not result in resolubilization of already precipitated proteins (Figure 1E). After all of these optimizations, we repeated our initial experiment using the quenching-based sample pooling. Figure 1F shows the volcano plot of a thus-obtained I-PISA data set from the MTX-treated K562 cell lysate. As in the initial I-PISA approach where the individual ion concentrations were diluted prior to pooling (Figure 1C), the optimized experiment shows a significant increase in DHFR solubility, confirming the viability of the quenching-based approach. The optimized I-PISA procedure thus looks as shown in Figure 1G. We used this optimized procedure in the rest of the study.

### I-PISA Assay for Multitarget Drugs

**Figure 2:**
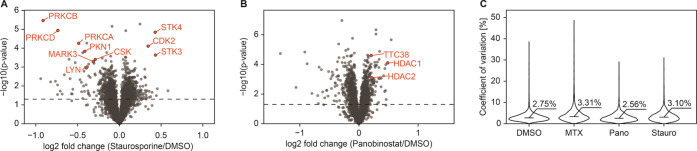
I-PISA assay in the lysate allows one to detect direct targets of multitarget drugs. (A) Treatment of the K562 lysate with 10 μM Staurosporine resulting in solubility change of several kinases. (B) I-PISA of the K562 lysate treated with 10 μM Panobinostat revealing known drug targets. (C) Median coefficient of variation (CV) of protein signals between the replicates in all lysate I-PISA assays is below 4%, highlighting the excellent reproducibility of the method. Abbreviations: MTX, methotrexate; Pano, Panobinostat; Stauro, Staurosporine; 4817 proteins were quantified for each panel.

Next, aiming to test the optimized I-PISA assay for multitarget drugs, we treated the K562 lysate for 15 min with either 10 μM Staurosporine (Stauro) (a pan-kinase inhibitor)^35^ or Panobinostat (Pano) (a pan-HDAC inhibitor).^36^ The Staurosporine results expectedly revealed that most of the proteins with the largest solubility changes are kinases (Figure 2A). These include several known targets with increased solubility, such as STK3, STK4, CDK2, as well as kinases with decreased solubility, such as multiple members of the protein kinase C family, CSK, LYN, PKN1, and MARK3.^9,35^

In the case of Panobinostat, known detected targets showed an increase in solubility. These include HDAC1 and HDAC2, as well as the known Panobinostat off-target TTC38^37^ (Figure 2B).

Importantly, the median coefficient of variation (CV) values of protein signals among the replicates in I-PISA were below 4% for all drug treatments (Figure 2C), indicating excellent reproducibility of the optimized workflow.

### Comparison of TPP with IPP

**Figure 3:**
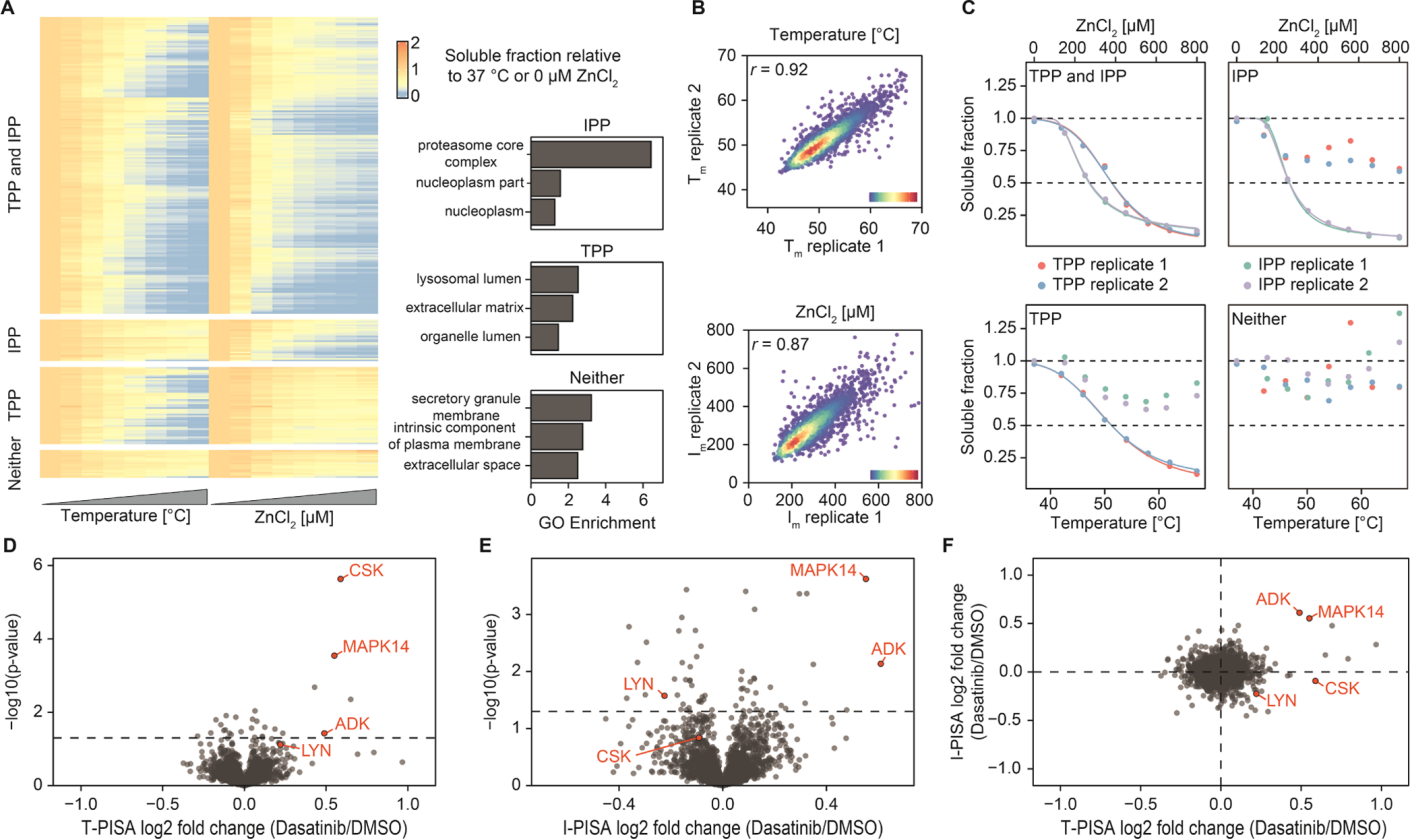
Comparison of temperature-based and ion-based protein precipitation. (A) Heatmap representing the average of two replicate precipitation curves for TPP and IPP relative to the solubility at the lowest temperature or ion concentration. The panel on the right shows Gene Ontology enrichment of high-quality curves either obtained exclusively by IPP or TPP or neither of the two. (B) Replicate-to-replicate repeatability of the *T*_m_ and *I*_m_, for TPP and IPP, receptively. (C) Examples of protein precipitation curves obtained by both methods (top left), only by IPP (top right), only by TPP (bottom left), or neither (bottom right). Comparison of T-PISA with I-PISA for the lysate treated with Dasatinib. (D) T-PISA and (E) I-PISA assay identifying direct binders of Dasatinib in the K562 lysate (2999 common proteins). (F) Direct comparison of solubility alteration with temperature and ion-based precipitation showing orthogonality of the two approaches to protein precipitation.

To assess the complementarity between temperature-based protein precipitation (TPP) and ion-based protein precipitation (IPP), we performed a melting curve experiment in the K562 cell lysate. Samples treated in two replicates at eight different temperatures or ZnCl_2_ concentration points were then digested and multiplexed individually into two TMTpro sets, each containing one replicate of both TPP and IPP. The TMTpro sets were separated into 12 fractions each and analyzed by LC–MS/MS. The resulting solubility curves were investigated, and the corresponding *T*_m_ and *I*_m_ values were determined following the TPP methodology.^9^ From the 5772 proteins reliability quantified across both TMTpro sets, a high-quality protein solubility curve (*R*^2^ > 0.8 and plateau < 0.3) could be fitted in both replicates for 5410 proteins for either TPP, IPP, or both (Figure 3A, left panel). Of these, 1008 (19% of all proteins with high-quality curves) or 541 (10%) curves were obtained exclusively for TPP or IPP, respectively. Interestingly, the proteins with exclusive melting curves showed significant enrichment of different Gene Ontology entries, supporting our initial hypothesis of different protein precipitation mechanisms (Figure 3A, right panel). Specifically, among the IPP-only proteins, the proteasome core complex is significantly enriched. Both precipitating approaches demonstrate excellent replicate-to-replicate repeatability for the conditions (temperature or ion concentration) at which half of the proteins are precipitated (Figure 3B). Examples of melting curves for both common and exclusive precipitations are given in Figure 3C.

### Comparison of T-PISA with I-PISA

To assess the degree of overlap and orthogonality between the targets identified by temperature-based PISA (T-PISA) and I-PISA, we treated the K562 lysate with Dasatinib, a tyrosine-kinase inhibitor,^38^ at a 10 μM concentration for 15 min and performed both T-PISA and I-PISA. As these analyses were made in four replicates each with multiplexing of all samples in one TMTpro set, the only difference we expected to see was due to the different protein precipitation approaches. The corresponding volcano plots for T-PISA and I-PISA are shown in Figure 3D,E, respectively. Direct comparison of the solubility shifts in Figure 3F shows that, while some proteins are significantly changing in the same direction in both methods, including known off-targets MAPK14 and ADK^2^ (Figure 3F), in general, there is no correlation (Pearson’s *r* = 0.06) between the TPP and IPP values. Importantly, some known Dasatinib targets, such as CSK and LYN^2^, are shifting exclusively in either temperature or ion-based precipitation. Also worth noticing is the opposite direction of solubility changes of LYN when engaging Dasatinib: while the protein solubility decreased in the ion-based method, it (or thermal stability) increased with temperature (Figure 3F). It is apparent from Figure 3C that the combined use of T-PISA and I-PISA may be beneficial in terms of both identifying more targets (when the target is highlighted by one of the techniques) as well as for increasing the certainty in target identification (when both techniques pinpoint the same target).

### I-PISA Assay in Living Cells

**Figure 4:**
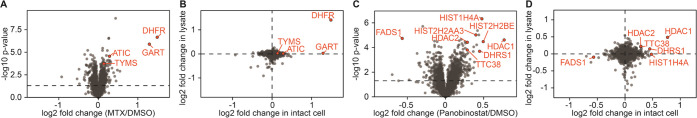
I-PISA assay in intact cells allows one to detect downstream effector proteins. (A) Volcano plot of K562 cells treated with MTX showing additional shifting proteins compared to the lysate experiment (4113 proteins quantified). (B) Comparison of protein solubility alterations between the lysate and cell experiments pinpointing downstream targets in the latter. (C) Treatment of K562 cells with Panobinostat highlighting downstream proteins of direct targets (4113 proteins quantified). (D) Comparison of I-PISA assay Panobinostat results obtained in cells versus lysates providing additional and/or more reliable target information.

Whereas solubility alteration experiments in cellular lysate provide information about direct binding events, experiments in intact cells additionally contain information about downstream interactions. After K562 cells were treated in growth media with MTX at a 2 μM concentration for 2 h, they were harvested into centrifugation tubes, treated with increasing ZnCl_2_ concentrations, and then lysed in the tubes by three freeze–thaw cycles. After that, the protein precipitation was quenched by Na_2_POH_4_, the samples were pooled together, and the precipitated proteins were removed by ultracentrifugation. The resulting volcano plot of 4113 proteins quantified with a median of eight peptides per protein is shown in Figure 4A. Not surprisingly, DHFR is shifted on that plot as a direct target of MTX, similar to lysate I-PISA (Figure 1F). However, direct solubility alteration comparison between the lysate and in-cell I-PISA assays in Figure 4B shows that such proteins from the mitochondrial folate-mediated one-carbon pathway as GART and ATIC, that are known to be downstream of DHFR in that pathway,^39^ are shifting only in intact cells. Interestingly, the thermal stability of these two proteins is not seen changing in temperature-based experiments performed on intact cells,^32,40^ which supports the orthogonality of ion-based and thermal protein precipitation approaches. Another known downstream target of MTX, thymidylate synthase (TYMS), shows a significant, albeit a rather small, alteration (Figure 4A), unlike in the lysate (Figure 4B). We also performed an in-cell I-PISA experiment with Panobinostat as an example of a multiple targeting drug (Figure 4C) and compared the in-lysate and in-cell results (Figure 4D). The overlapping proteins are the known direct targets HDAC1, HDAC2, and TTC38, while the proteins shifting only in cells are the known downstream proteins FADS1, DHRS1, and several histones.^9,37,41^

### Miniaturization of the I-PISA Assay

**Figure 5:**
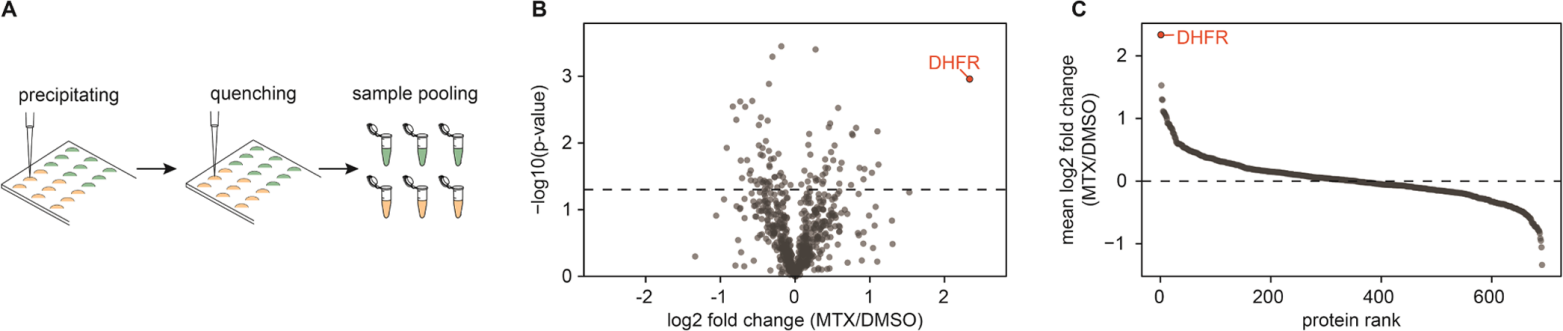
Miniaturization of the I-PISA assay. (A) Schematic illustration of the I-PISA experimental procedure for minimal sample volumes handled on microscope cover slides. (B) I-PISA volcano plot obtained from 1 μg of lysate treated with MTX detecting a significant increase in solubility of the known target DHFR among 691 quantified proteins. (C) Order of quantified proteins sorted by their mean log2-scaled fold change, highlighting the discrimination power of I-PISA for small-volume protein samples.

With the advent of single-cell proteomics, the interest in analysis of small proteome samples has greatly increased.^42,43^ T-PISA has much smaller sample requirements than TPP, and yet, its sample demand greatly exceeds the protein amount that can be extracted from a single cell. One of the main limitations is that temperature-based protein precipitation requires a sufficiently high protein concentration for aggregation of proteins. Additionally, the employment of a thermal cycler in T-PISA requires the usage for protein precipitation of PCR tubes that possess a relatively high volume and are not amenable to miniaturization. Contrary to that, I-PISA does not require any specific vessel and ion-based precipitation could be less sample demanding than thermal-based methods. To test this latter hypothesis, we performed an I-PISA experiment with the K562 lysate treated with 10 μM MTX using only 1 μg of protein lysate per replicate (an equivalent of 7736 ± 518 cells, Figure S2). To keep the protein concentration high, we adopted from single-cell proteomics the concept of nanoPOTS.^44^ The entire I-PISA sample preparation was performed on microscope cover slides, allowing minimal working volume with limited protein losses (Figure 5A). Despite the small sample amount, I-PISA identified DHFR as the sole direct target of MTX (Figure 5B), discriminating it from other 691 quantified proteins with high specificity (Figure 5C).

## Conclusions

Here, we present a method of fast isothermic protein precipitation based on the kosmotropic effect of ions from the Hofmeister series. The developed I-PISA method can assess solubility changes in both cell lysates and intact cells. It also allows for pooling samples together before centrifugation for reducing labor costs and increasing throughput. Importantly, I-PISA shows complementary solubility shifts (and in some cases shifts in the opposite direction) to thermal proteome profiling.

We also demonstrated that the I-PISA approach works for limited sample amounts that are smaller than currently amenable to T-PISA or TPP. Therefore, being competitive with thermal proteome profiling, I-PISA can be a valuable addition to the arsenal of chemical proteomics tools probing small-molecule engagement by proteins, such as targets and off-targets in drug development. Besides that, observation in intact cells of the solubility shifts in proteins downstream of direct targets demonstrates that I-PISA can be used for general profiling of changes in protein primary, secondary, and higher-order structures. A potential limitation of I-PISA is when the test molecules are chelating the metal ions used, interfering with protein precipitation. This for example could be the case when determining the proteome-wide binding landscape of nucleotides, the negatively charged molecules known to chelate zinc cations. However, such a potential problem can be solved by “salting-out” anions, such as HCO_3_^–^, e.g., in the form of NaHCO_3_ salt (Figure 1A). Compared to TPP, IPP data sets appear to be enriched with high-quality data for proteins belonging to the proteasome and nucleoplasm, representing a promising class of drug targets.^45^ Thus, the ion-based protein precipitation approach offers novel possibilities for finding drug targets and interacting partners in both lysates and intact cells. With the advent of complementary TPP protein precipitation approaches, such as solvent, mechanic, and ion-based protein precipitation, the number of false-negative identifications (missed drug targets) can be reduced, making these techniques even more valuable in drug development. We hypothesize that I-PISA will be widely used, possibly as a PISA complement, in drug discovery, molecular biology, personalized medicine, and stem cell research.

## Supporting Information Available

Detailed description of experimental results and methods including cell culture, cell lysate preparation, proof-of-concept experiments, proteomic sample preparation, setting for LC–MS/MS, data analysis for PISA, and curve fitting for the TPP/IPP experiment (PDF)
Statistical analysis of all PISA experiments (XLSX)
Data for TPP and IPP curve fitting (XLSX)
