# Supplementary material for: Ion-Based Proteome-Integrated Solubility Alteration Assays for Systemwide Profiling of Protein–Molecule Interactions
Source: Anal Chem. 2022 May 4;94(19):7066–74. doi: 10.1021/acs.analchem.2c00391 (PMC9118197; doi:10.1021/acs.analchem.2c00391)
Supplement: Supplementary file 1 — ac2c00391_si_001.pdf [file ac2c00391_si_001.pdf]

SUPPORTING INFORMATION

Ion-based Proteome Integrated Solubility Alteration Assay for System-wide Profiling of Protein-Molecule Interactions

Christian M. Beusch[1], Pierre Sabatier[1], Roman A. Zubarev[1,2,3]*

Affiliations

[1] Chemistry I, Department of Medical Biochemistry and Biophysics, Karolinska Institute, Stockholm 17177, Sweden

[2] Department of Pharmacological & Technological Chemistry, I.M. Sechenov First Moscow State Medical University, Moscow 119146, Russia

[3] The National Medical Research Centre for Endocrinology, Moscow 115478, Russia

*Corresponding authors. Email: roman.zubarev@ki.se

Table of Contents

Supplementary Figures S1-S2 (page S2-3)

Extended Materials and Methods (pages S4-S7)

- Cell culture (page S4)
- Harvest cells (page S4)
- Proof of principle samples with BCA readout (page S4)
- Proteomic sample preparation (page S4-S5)
- Offline high pH reverse-phase fractionation (page S5)
- LC-MS/MS (page S5-S6)
- Data Processing and Statistical Analyses (page S7)
- Gene Ontology enrichment analysis (page S7)

References (page S7)

Supplementary Figures

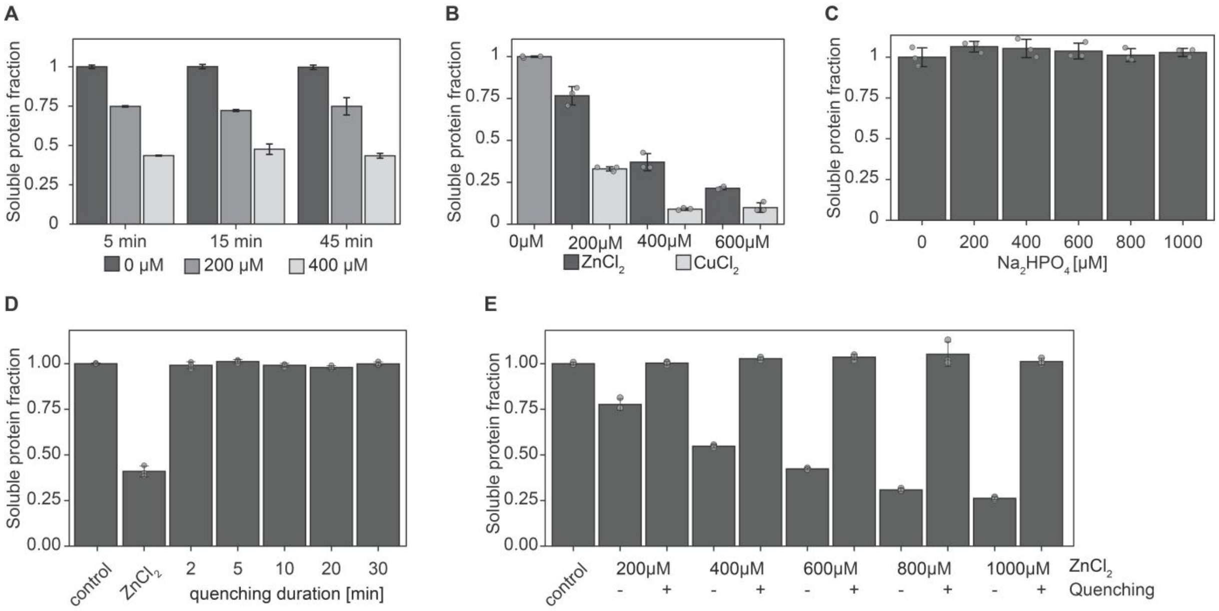

Figure S1. a) Protein precipitation by $CuCl_2$ is time independent. b) Protein precipitation with $CuCl_2$ is more abrupt compared to $ZnCl_2$. c) Incubation of protein lysate with an increasing concentration of Na_2HPO_4 does not result in protein precipitation. d) The quenching action of 1mM $ZnCl_2$ is almost immediately on a minute scale. e) Quenching of different concentrations of $ZnCl_2$ for the entire concentration range.

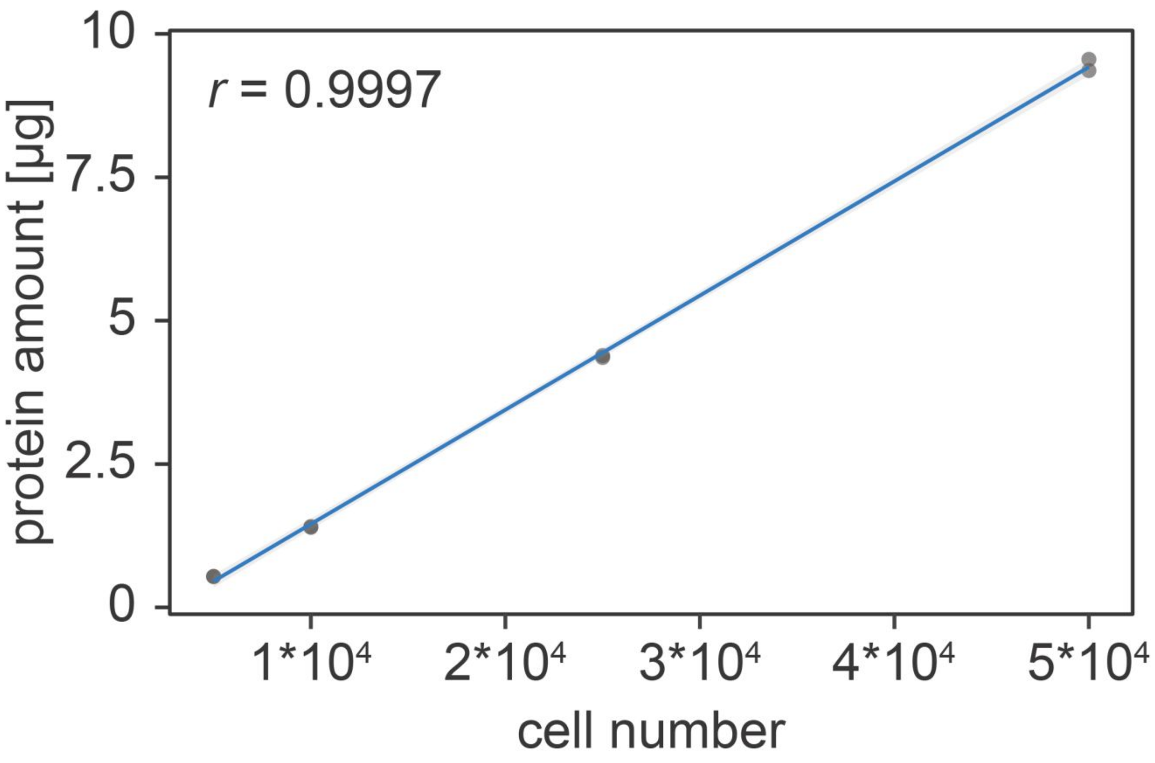

Figure S2. Determination by BCA of protein amount in a lysate obtained from small numbers of K562 cells by freeze-thaw.

Experimental procedures

Cell culture

K562 and A549 cells were cultured at 37 °C and 5% CO_2 in a humidified Forma Steri-Cycle i160 CO_2 incubator (Thermo Fischer Scientific). They were maintained in Iscove's Modified Dulbecco's Medium (Biowest) or Dulbecco's Modified Eagle Medium (Lonza), respectively, supplemented with 10% FBS (Gibco, Invitrogen) and 100 units/mL penicillin/streptomycin (Gibco, Invitrogen). The DMEM media was additionally supplemented with 2 mM L-glutamine (Lonza). For all experiments, cell lines were kept for up to 10 passages for K562 and 13 passages for A549 cell lines.

Harvest cells

A549 cells were harvested when reaching approximately 80% confluency, washed two times with 50 mM HEPES buffer containing 150 mM NaCl at pH 7.4 and detached by TrypLE (Gibco). Cells were centrifuged and resuspended in 50 mM HEPES buffer containing 150 mM NaCl at pH 7.4. The washing step was repeated twice. K562 cells were cultured in 175 cm^2 flasks up to $1.5*10^6$ cells/mL and washed 2 times with 50 mM HEPES buffer containing 150mM NaCl at pH 7.4 by centrifuging for 3 min at 1000 rpm. The washed cell pellets were resuspended in the same buffer containing Halt protease inhibitor (Thermo Fisher Scientific) according to the manufacturer's instructions. For the full curve proteomics experiment, K562 cells were washed as in other experiments but lysed by three cycles of freeze-thawing in liquid nitrogen in 50 mM HEPES buffer containing 150 mM NaCl and 0.4% NP-40 at pH 7.4 and Halt protease inhibitor (Thermo Fisher Scientific). For experiments where cell lysate was required, cells were lysed by three freeze-thawing cycles in liquid nitrogen, followed by centrifugation for 10 min at 21'000 g at 4 °C to collect the soluble protein content. The protein amount was determined by BCA assay (Thermo Fisher Scientific) according to the manufacturer's instructions and diluted to 1 mg/mL for all experiments unless otherwise stated and stored at -80 °C.

Proof of principle samples with BCA readout

The cell lysate was treated with the corresponding salt concentration for 10 min at room temperature if not otherwise specified. Quenching with a specified amount of Na_2HPO_4 for 10 min when stated. Aggregated proteins were removed by centrifugation at 17'000 g for 10 min. The supernatant was collected, and protein concentration was determined by BCA assay (Thermo Fisher Scientific).

Proteomic sample preparation

A defined amount of soluble proteins was incubated at a final concentration of 4 M Urea and 5 mM Dithiothreitol for 1 h at room temperature, followed by alkylation using 15 mM Iodoacetamide for 1 h in the dark. Thereafter, proteins were precipitated by chloroform/methanol precipitation. In brief, 4 volumes (relative to the samples) of methanol were added to the samples, followed by 1 volume of chloroform, and 3 volumes of water. Samples were vortexed and centrifuged at 20'000 g for 10 min at 4 °C. The organic phase was removed, and the protein pellet was rinsed with 1 volume of methanol before the samples were centrifuged again. All liquid was removed, and samples were let to dry until appearing whitish. Samples were resuspended in 20 mM EPPS at pH 8.5 and 8 M Urea, then the Urea concentration was diluted to 4 M and Lysyl Endopeptidase (Wako, Fujifilm) was added (1:100 w/w) and incubated for 2 h. Then

samples were diluted to 1 M Urea and Trypsin (Promega) was added and incubated overnight at room temperature. TMT-10plex or TMTpro-16plex (Thermo Fischer Scientific) reagents were resuspended into dry ACN and added to each sample with four times excess relative to protein amount. The ACN amount in the samples was adjusted to 20 % and samples were incubated for 2 h at room temperature. Labeling was quenched by adding hydroxylamine (Thermo Fischer Scientific) to a final concentration of 0.5% and incubated for 15 min. Samples were combined, ACN was evaporated in the SpeedVac (Genevac), samples were acidified with TFA pH < 3 and were desalted by SepPack (Waters).

Offline high pH reverse-phase fractionation

For offline high pH reverse-phase fractionation, the sample was resuspended in 20 mM NH_4OH and fractionation was performed using an UltimateTM 3000 RSLCnano System (Dionex) equipped with an XBridge Peptide BEH 25 cm column of 2.1 mm internal diameter, packed with 3.5 µm C18 beads having 300 Å pores (Waters). The mobile phase consisted of buffer A (20 mM NH_4OH in miliQ water) and buffer B (20 mM NH_4OH in ACN). The gradient started from 1% B to 23.5% in 42 min, then to 54% B in 9 min, 63% B in 2 min and stayed at 63% B for 5 min and finally back to 1% B and stayed at 1% B for 7 min. This resulted in 96 fractions that were concatenated according to the experimental plan, dried in a SpeedVac (Genevac) and stored at -80 °C.

LC-MS/MS

Prior to mass-spectrometry analysis, the samples were resuspended in 2% ACN and 0.1% FA (Buffer A) and injected into an UltiMate 3000 UPLC autosampler or EASY-LC (Thermo Scientific Scientific). The peptides were loaded on a trap column (Acclaim PepMap 100 C18, 100 µm × 2 cm) and separated on a 50 cm long C18 Easy spray column (Thermo Scientific Scientific). The chromatographic gradients and the mass spec settings were as follows:

	I-PISA experiments in cell and lysate	I- and T-PISA comparison experiment	I-PISA with equilibration	I-PISA with 1 µg starting material	Full curve experiment
LC system	UltiMate 3000 UPLC	UltiMate 3000 UPLC	EASY-LC	UltiMate 3000 UPLC	UltiMate 3000 UPLC
Gradient time	110 min	120 min	120 min	135 min	120 min
Gradient steps	0-3 min 4%, 75 min 26%, 95 min 35%,	0-5 min 4%, 90 min 26%, 105 min	0-3 min 5%, 93 min 28%, 103 min	0-3 min 4%, 100 min 24%,	0-3 min 4%, 80 min 24%, 105 min

	98 min 95%, 103 min 95%, 105 min 4%	35%, 108 min 95%, 114 min 95%, 115 min 4%	95%, 110 min 95%, 112 min 5%	120 min 32%, 122 min 95%, 127 min 95%, 128 min 4%	35%, 108 min 95%, 113 min 95%, 115 min 5%
Mass Spectrometer	Fusion Lumos	Fusion	QE	Fusion Lumos	Fusion Lumos
Scan cycle, s	3	2	Loop 15	-	3
MS1 resolution	120’000	120’000	120’000	120’000	120’000
MS1 m/z range	375-1400	375-1400	375-1500	390-1100	375-1400
Injection time, ms	50	50	120	50	50
MS1 AGC	$1*10^6$	$1*10^6$	$1*10^6$	$1*10^6$	$1*10^6$
Included charge states	2-6	2-6	2-6	-	2-6
Exclusion duration, s	45	45	90	-	45
Isolation window, m/z units	0.7	0.7	1.2	24	0.7
NCE, %	35	35	32	26, stepped 2	35
MS2 resolution	50’000	50’000	35’000	30’000	50’000
Max injection time, ms	86	86	120	54	86
MS2 AGC	$2.5*10^5$	$2.5*10^5$	$2*10^5$	$4*10^5$	$2.5*10^5$

Data Processing and Statistical Analyses
All downstream analysis of proteomics data was done in R (version 4.1.1). From the MaxQuant results, known contaminants, decoy proteins, as well as proteins with fewer than 2 peptides were excluded from further analysis. Protein abundances were normalized by Variance Stabilizing Normalization[1] and statistical differences between the groups were calculated by a two-tailed t-test with equal variances. Fold changes for all PISA experiments were calculated based on generalized log-scaled abundances of the TMT reporter ions.

For the analysis of the TPP and IPP datasets, the previously developed SIESTA R package was adopted [2]. In short, after removing proteins with missing values (i.e., not quantified in all samples), protein solubility curves were scaled to the lowest temperature or Zinc concentration for TPP and IPP, respectively. In order to account for pipetting variation, protein precipitation curves were normalized as in TPP [3,4]. In brief, for all samples proteins with the 4th point between 0.4 and 0.6, 6th point below 0.3 and 8th point below 0.2 were used to generate a normalization curve. The normalized data were then fitted to a sigmoidal curve and their T_m or I_m was determined as the temperature or ion concentration, at which half of the proteins are soluble. Protein curves with $R^2 > 0.8$ and a plateau of < 0.3 were classified as high-quality precipitation curves.

Gene Ontology enrichment analysis
Gene Ontology (GO) enrichment for the proteome profiling experiment was performed with Gorilla [5]. All reliably quantified proteins from the TPP and IPP experiment were used as a background. The three most significant Cellular component GO terms from each analysis were selected as the output.

References

[1] W. Huber, A. Von Heydebreck, H. Sültmann, A. Poustka, M. Vingron, *Bioinformatics* **2002**, *18*, S96–S104.

[2] A. A. Saei, C. M. Beusch, P. Sabatier, J. A. Wells, H. Gharibi, Z. Meng, A. Chernobrovkin, S. Rodin, K. Näreoja, A.-G. Thorsell, T. Karlberg, Q. Cheng, S. L. Lundström, M. Gaetani, Á. Végvári, E. S. J. Arnér, H. Schüler, R. A. Zubarev, *Nat. Commun. 2021 121* **2021**, *12*, 1–13.

[3] H. Franken, T. Mathieson, D. Childs, G. M. A. Sweetman, T. Werner, I. Tögel, C. Doce, S. Gade, M. Bantscheff, G. Drewes, F. B. M. Reinhard, W. Huber, M. M. Savitski, *Nat. Protoc. 2015 1010* **2015**, *10*, 1567–1593.

[4] M. M. Savitski, F. B. M. Reinhard, H. Franken, T. Werner, M. F. Savitski, D. Eberhard, D. M. Molina, R. Jafari, R. B. Dovega, S. Klaeger, B. Kuster, P. Nordlund, M. Bantscheff, G. Drewes, *Science (80-.).* **2014**, *346*, 1255784.

[5] E. Eden, R. Navon, I. Steinfeld, D. Lipson, Z. Yakhini, *BMC Bioinformatics* **2009**, *10*, 1–7.
